# Learning to like disgust: neuronal correlates of counterconditioning

**DOI:** 10.3389/fnhum.2013.00346

**Published:** 2013-07-08

**Authors:** Jan Schweckendiek, Tim Klucken, Christian J. Merz, Sabine Kagerer, Bertram Walter, Dieter Vaitl, Rudolf Stark

**Affiliations:** ^1^Department of Psychotherapy and Systems Neuroscience, Justus Liebig University GiessenGiessen, Germany; ^2^Department of Cognitive Psychology, Institute of Cognitive Neuroscience, Ruhr-University BochumBochum, Germany; ^3^Bender Institute of Neuroimaging, Justus Liebig University GiessenGiessen, Germany

**Keywords:** counterconditioning, classical conditioning, evaluative conditioning, fMRI, disgust, reward learning

## Abstract

Converging lines of research suggest that exaggerated disgust responses play a crucial role in the development and maintenance of certain anxiety disorders. One strategy that might effectively alter disgust responses is counterconditioning. In this study, we used functional magnetic resonance imaging (fMRI) to examine if the neuronal bases of disgust responses are altered through a counterconditioning procedure. One disgust picture (conditioned stimulus: CS+_disg_) announced a monetary reward, while a second disgust picture (CS-_disg_) was never paired with the reward. Two neutral control pictures (CS+_con_/CS-_con_) were conditioned in the same manner. Analyses of evaluative conditioning showed that both CS+ were rated significantly more positive after conditioning as compared to the corresponding CS−. Thereby, the CS+_disg_ and the CS+_con_ received an equal increase in valence ratings. Regarding the fMRI data, ANOVA results showed main effects of the conditioning procedure (i.e., CS+ vs. CS−) in the dorsal anterior cingulate cortex. Further, main effects of the picture category (disgust vs. control) were found in the bilateral insula and the orbitofrontal cortex. No interaction effects were detected. In conclusion, the results imply that learning and anticipation of reward was not significantly influenced by the disgust content of the CS pictures. This suggests that the affect induced by the disgust pictures and the affect created by the anticipation of reward may not influence the processing of each other.

## Introduction

A growing line of evidence suggests that the emotion disgust plays an important role in the etiology and maintenance of psychiatric disorders like obsessive-compulsive disorder, specific phobias, eating disorders, and even post-traumatic stress disorder (Olatunji et al., 2010; Mason and Richardson, 2012). The emotion disgust has unique features and has been shown to be very resistant to extinction (Rozin and Fallon, 1987; Olatunji et al., 2010; Mason and Richardson, 2012). This may in part explain the difficulties in the treatment of these disorders (Mason and Richardson, 2012) with exposure therapy, which is based on extinction (e.g., McNally, 2007). Insight into the neuronal circuitry underlying the alteration of disgust responses can be used to improve treatment strategies. In the present study, we investigated if subjective and hemodynamic disgust responses are altered through a counterconditioning procedure.

Although counterconditioning has been examined in some detail in animals (e.g., Dickinson and Pearce, 1977; Bouton, 2004), human studies are sparse. This is surprising, since many influential theories of reinforcement learning make explicit predictions for counterconditioning (e.g., Dickinson and Pearce, 1977 see Daw et al., 2002). Counterconditioning describes the process in which a CS is first paired with one unconditioned stimulus (UCS) and then paired with another UCS of incompatible affective value in a second step (Bouton, 2004). However, in some experimental designs counterconditioning refers merely to the pairing of stimuli of opposing valence (cf. Jong et al., 2000). This was also the case in the present study, in which disgust inducing stimuli were paired with an appetitive reward stimulus. One recent study was able to show that counterconditioning of conditioned disgust-related evaluative responses was more effective compared to extinction as measured by pleasantness ratings and an affective priming task (Kerkhof et al., 2011). Moreover, counterconditioning has been found to improve exposure therapy in spider phobics with regard to valence and fear ratings as well as heart rate changes (Eifert et al., 1988 but see Jong et al., 2000). Although both studies did not directly measure disgust ratings, the results suggest that counterconditioning can modify evaluative responding to disgust stimuli, at least in terms of valence. However, no study to date has examined the underlying neuronal mechanisms.

Given that counterconditioning is able to change responding to disgust stimuli, this should result in an alteration of brain activity in areas that have been associated with disgust processing. The insula plays a central role in disgust processing and the recognition of disgust from facial expressions. Enhanced insula reactions were observed in response to disgust inducing pictures and video clips in a variety of studies (e.g., Schienle et al., 2002b; Wright et al., 2004; Caseras et al., 2007; Jabbi et al., 2008; Schäfer et al., 2009). Moreover, insula activity is correlated with the subjective experience of disgust (Fitzgerald et al., 2004; Stark et al., 2007) and the personality trait disgust sensitivity (Calder et al., 2001; Schienle et al., 2003; Stark et al., 2005; Caseras et al., 2007; Schäfer et al., 2009; Olatunji et al., 2010; Klucken et al., 2012a). In addition to the insula, converging evidence points to the amygdala, the orbitofrontal cortex (OFC), and the dorsal striatum as important structures in disgust processing (Calder et al., 2001; Phan et al., 2004; Vytal and Hamann, 2010).

Moreover, because counterconditioning of disgust stimuli entails aspects of reward learning and anticipation, it could also affect areas related to these processes. Studies in animals and humans have implicated the ventral striatum [especially the nucleus accumbens (NAcc)], the OFC, the amygdala, the dorsal anterior cingulate cortex (dACC), and the insula (Martin-Soelch et al., 2007; Klucken et al., 2009b; Haber and Knutson, 2010; Klucken et al., 2012b). Activity of the NAcc and the OFC has been reported to shift from the onset of the UCS to the onset of the CS as the occurrence of the UCS becomes more predictable during the course of conditioning (Schultz, 1997; McClure et al., 2003; O'Doherty et al., 2003). The amygdala has been consistently implicated in animal studies of reward learning and anticipation (Haber and Knutson, 2010), however, only few human studies have reported an involvement of the amygdala (e.g., Gottfried et al., 2002). In addition, studies using monetary reward as UCS have reported dACC and insula activation (Kirsch et al., 2003; Cox et al., 2005). The dACC has not only been consistently implicated in reward-related learning and anticipation, but also in tasks that require error detection, response override, and other forms of conflict including emotional conflict (for review see Botvinick, 2007; Carter and van Veen, 2007; Taylor et al., 2007). Moreover, it has been proposed that the dACC and the insula form the core of a salience network, which is activated in response to important environmental stimuli (Menon and Uddin, 2010).

In the present study, we tested whether activity of the mentioned brain regions is altered by a counterconditioning procedure. To this end, we designed a novel paradigm using a classical conditioning approach. In a differential conditioning design, one disgust picture (CS+_disg_) predicted a monetary reward (UCS), while a second disgust picture (CS−_disg_) was never paired with the reward. A second pair of neutral control pictures (CS+_con_/CS−_con_) served as a control condition and was differentially conditioned in the same manner, again using monetary reward as UCS. Using this 2 (CS-emotion: disgust vs. control) × 2 (reward learning: CS+ vs. CS−) factorial design allowed us to investigate the effect of the counterconditioning procedure while controlling for mere effects of the emotional content of the pictures and the effects of reward learning and anticipation (i.e., of conditioning). In accordance with previous studies, we hypothesized that the counterconditioning procedure would shift subjective valence ratings of the CS+_disg_ in the positive direction. Regarding hemodynamic responses, we expected the CS+_disg_ to elicit altered activity of structures related to the processing of disgust responding and of structures related to reward learning and anticipation as compared to the CS−_disg_. In detail, we expected enhanced responses of the dACC, the insula, the NAcc, the amygdala, and the OFC as a correlate of the counterconditioning procedure.

## Methods and materials

### Subjects

Thirty-two healthy (16 female, 16 male) subjects were recruited from campus advertisements; four subjects were excluded from analyses because of extensive head movement, drowsiness during scanning (two subjects), and an extremely low disgust sensitivity score (i.e., >2 standard deviations below the group mean; Schienle et al., 2002a) leaving 28 subjects in the final sample (12 male, 16 female; *M*_age_ = 25.93; *SD*_age_ = 3.22). All subjects were students at the Justus Liebig University Giessen, right-handed, and had normal or corrected-to-normal vision. No subject had ever received psychotropic medication or psychotherapeutic treatment. Participants were informed about the procedure in general and gave written informed consent. All experimental procedures were in accordance with the Declaration of Helsinki and were approved by the local ethics committee of the Institute for Psychology and Sports Science at the Justus Liebig University Giessen.

### Stimuli

Two pictures of disgust scenes (dirty toilets) and two pictures of household items (a dish and a stool) served as CS in the experimental condition. Two pictures were taken from the International Affective Pictures System (Lang et al., 2008; picture numbers: 7006, 9300), the other two were collected by the authors. All pictures had been successfully used in previous studies (Stark et al., 2004, 2007). An amount of 0.50€ was used as UCS, which was represented by a cartoon drawing of coin stacks collected by the authors. Pictures were comparable with regard to complexity as far as possible in order to prevent confounding effects. Stimuli were projected onto a screen at the end of the scanner (visual field = 18°) using an LCD projector (EPSON EMP-7250) and were viewed through a mirror mounted on the head coil.

### Procedure

Subjects were instructed that they would be exposed to emotionally disgust scenes and pictures of everyday items. Further, subjects were told that they would receive 15€ for participation and an additional amount of 0.50€ for each time they saw the picture of the coin stacks at the end of the experiment and that they didn't have to do anything to obtain the money. Moreover, subjects were instructed to look at the pictures and to pay attention to possible relationships between the monetary reward and the other pictures presented during the experiment (cf. Schiller et al., 2008; Raes et al., 2009; Schweckendiek et al., 2011; Klucken et al., 2012a).

The classical conditioning design was adopted from previous studies using pictures as UCS (e.g., Klucken et al., 2009a,b; Schweckendiek et al., 2011). During the experiment subjects passively viewed the images while hemodynamic responses were recorded. Except for the subjective ratings (see below), no other behavioral measures were collected. The experiment consisted of a habituation phase, a conditioning phase, and an extinction phase. During the habituation phase each of the four CS pictures was presented 10 times. One trial consisted of the presentation of a CS picture for 3 s followed by the inter trial interval (ITI), which ranged from 3 to 8 s (see below). During the conditioning phase each CS was presented 16 times for 8 s. One disgust (CS+_disg_) and one neutral picture (CS+_con_) were followed by the picture of coin stacks (UCS) that represented the gain of 0.50€ for 3 s with 100% reinforcement with no delay, while the remaining disgust (CS−_disg_) and neutral (CS−_con_) pictures were never followed by the UCS. The ITI again ranged from 3 to 8 s (see below). During the extinction phase, all CS were again presented 10 times each for 3 s. One trial again consisted of the presentation of a CS picture and the ITI, which ranged from 3 to 8 s (see below). In total, subjects were exposed to each picture for 30 s (10 × 3 s) during the habituation and for 128 s (16 × 8 s) during the conditioning phase. The short extinction phase is part of another project and will not be discussed here in detail. Only data from the habituation and the conditioning phase are reported in this manuscript.

Stimulus allocation as CS+ and CS− was counterbalanced between participants. The ITI was equally distributed between 3 and 8 s. A small fixation cross was presented at the center of the screen during the ITI. ITIs were calculated to contain equally distributed stimulus-onset-asynchronies (ranging from 0 to 2.5 s) in order to optimize signal acquisition for the whole-brain. Stimuli were presented in a pseudo-randomized order with the restrictions: (1) no more than two consecutive presentations of the same CS, (2) no more than two consecutive presentations of the same CS-type (i.e., CS+/CS−), (3) no more than two consecutive presentations of the same picture category (i.e., disgust/control), and (4) an equal quantity of each CS within the first and the second half of the conditioning phase. Throughout the experiment an MRI-compatible video camera was used to insure that subjects watched the stimuli. After the experiment, participants filled out the Questionnaire for the Assessment of Disgust Sensitivity (QADS; Schienle et al., 2002a) assessing individual proneness to disgust. The questionnaire was designed based on the questionnaire by Haidt et al. (1994) and describes 37 situations, which have to be judged on a five point scale regarding their ability to induce disgust. The questionnaire consists of five different subscales: (1) death/deformation (2) body secretion (3) spoilage/decay (4) poor hygiene (5) oral rejection. Cronbach's α of the total scale is 0.90 with the subscales varying between 0.69 and 0.85.

### Subjective ratings of the CS

In addition to the neuroimaging data, subjective ratings of the stimuli were collected. Before the habituation phase and after the conditioning phase subjects rated valence, arousal, and disgust for each of the four CS (CS+_disg_; CS−_disg_; CS+_con_; CS−_con_) on a nine-point Likert scale ranging from 1 (“very unpleasant”; “calm and relaxed”; “not disgusting at all”) to 9 (“very pleasant”; “very arousing”; “very disgusting”). Arousal and disgust ratings were collected on an exploratory basis. The measurement of subjective ratings before and after conditioning ensured that differences in the ratings were due to the counterconditioning procedure, while controlling for pre-existing differences and effects of the repeated presentation of the stimuli. The assessment of subjective ratings before the habituation phase ensured a relatively unbiased evaluation of the stimuli. Statistical analyses of the ratings were performed by means of a 2 × 2 × 2 ANOVA with the within-subject factors “reward learning” (CS+ / CS−), “phase” (habituation phase, conditioning phase) and “CS-emotion” (disgust/control) as implemented in SPSS 19 (IBM Corporation, Armonk, NY, USA) separately for each of the three rating dimensions (valence, arousal, disgust).

### Magnetic resonance imaging

Functional and anatomical scans were obtained using a 1.5 T whole-body tomography (Siemens Symphony) with a standard head coil. Structural image acquisition consisted of 160 T1-weighted sagittal images (MPRage, 1 mm slice thickness). A gradient echo field map was acquired before the functional image acquisition to obtain information for unwarping B_0_ distortions. For functional imaging a total of 832 volumes were recorded using a T2^*^-weighted gradient echo-planar imaging sequence (EPI) with 25 slices covering the whole-brain (slice thickness = 5 mm; gap = 1 mm; descending slice order; *TA* = 100 ms; *TE* = 55 ms; *TR* = 2.5 s; flip angle = 90°; field of view = 192 × 192 mm; matrix size = 64 × 64 pixel). The orientation of the axial slices was tilted 30° to the AC-PC line to keep susceptibility artifacts in the ventromedial parts of the frontal cortex to a minimum (cf. Deichmann et al., 2003; Weiskopf et al., 2006). Functional data were analyzed for outlying volumes using a distribution free approach for skewed data: outlier detection was based on a comparison of each volume with its two neighbors in a motion corrected time series. This was done by calculating the mean squared differences to the previous and the next volume. The smaller difference was used as deviation score for each volume. The scores were thresholded using the method of Hubert and van der Veeken (2008). Each resulting outlying volume was later modeled within the general linear model (GLM) with a covariate of no interest.

Preprocessing and statistical analyses were performed using Statistical Parametric Mapping (SPM8, Wellcome Department of Cognitive Neurology, London, UK; 2009) implemented in Matlab R2007b (Mathworks Inc., Sherborn, MA). Preprocessing of functional data included unwarping and realignment to the first volume (b-spline interpolation), slice time correction, normalization to the standard space of the Montreal Neurological Institute brain (MNI-brain) and smoothing with an isotropic three dimensional Gaussian kernel with a full-width-at-half-maximum (FWHM) of 9 mm.

Following experimental conditions were modeled in the general linear model for each subject: CS+_disg_ (paired disgust CS), CS−_disg_ (unpaired disgust CS), CS+_con_ (paired control CS), CS−_con_ (unpaired control CS) separately for the different phases of the experiment, UCS and non-UCS (i.e., the time after the CS-corresponding to the time of the UCS presentation after the CS+; Klucken et al., 2009a,b; Merz et al., 2010). In addition, the rating phases were modeled as nuisance regressors. The experimental conditions were modeled by stick functions convolved with the canonical hemodynamic response function. The six movement parameters obtained by the realignment procedure as well as the outlying volumes were introduced as covariates in the model. Additionally, a high pass filter (time constant = 180 s) was implemented using discrete cosine functions regressors. The subject level models were estimated after pre-whitening.

Beta-estimates of each regressor were calculated for each individual and were introduced as dependant variables to the second level random effects group analyses. Because individual disgust sensitivity is known to influence neuronal activity in response to disgust stimuli (Calder et al., 2001; Schienle et al., 2003; Stark et al., 2005; Caseras et al., 2007; Schäfer et al., 2009; Olatunji et al., 2010; Klucken et al., 2012a,b), DS scores were introduced as covariate of no interest to all contrasts involving the disgust pictures. DS scores were also correlated (voxel-wise simple regression) with the contrast CS+_disg_ vs. CS−_disg_. As a manipulation check, we first compared the UCS to the non-UCS in the conditioning phase and, in addition, the disgust to the control pictures in the habituation phase using paired *t*-tests.

To analyze main and interaction effects during the conditioning phase a 2 (“CS-emotion”: disgust vs. control) × 2 (“reward learning”: CS+ vs. CS−) full factorial model (Penny and Henson, 2007) was used in order to avoid potentially biased Type I errors in second level analyses due to the use of pooled errors (Boik, 1981; Barcikowski and Robey, 1984). Further we compared CS+_disg_ vs. CS−_disg_, as well as CS+_con_ vs. CS−_con_ using one-sample *t*-tests. Moreover, in order to link the hemodynamic responses to evaluative conditioning, we correlated (voxel-wise simple regression) the mean differential valence scores with the contrasts CS+_disg_ vs. CS−_disg_ and CS+_con_ vs. CS−_con_.

Within all models, we first performed explorative whole-brain analyses (*p*_FWE_ < 0.05 corrected for the whole-brain; Worsley, 2007). The next step was to test our a priori regions of interest (ROI) using the small volume correction feature of SPM (*p*_FWE_ < 0.05 corrected for search volume; (Worsley, 2007); cluster forming threshold: *p*_uncorr_< 0.001, *k*>5). The ROI analyses were performed for the following structures: insula, dACC, NAcc, amygdala, OFC, and dorsal striatum. All masks were created from the probabilistic Harvard-Oxford Cortical and Subcortical Atlases (included in FSLView version 3.1; http://www.fmrib.ox.ac.uk/fsl/; cf. Schweckendiek et al., 2011). Anatomical labeling of the exploratory whole-brain analyses was also performed using the Harvard-Oxford Cortical and Subcortical Atlases. The significance threshold was set to α = 0.05 corrected for multiple testing using family-wise-error correction as implemented in SPM8

## Results

### Analyses of subjective ratings

Subjective valence, arousal, and disgust ratings were analyzed separately using a 2 (“reward learning”: CS+ vs. CS−) × 2 (“phase”: habituation vs. conditioning) × 2 (“CS-emotion”: disgust vs. control) ANOVA.

**Figure 1:**
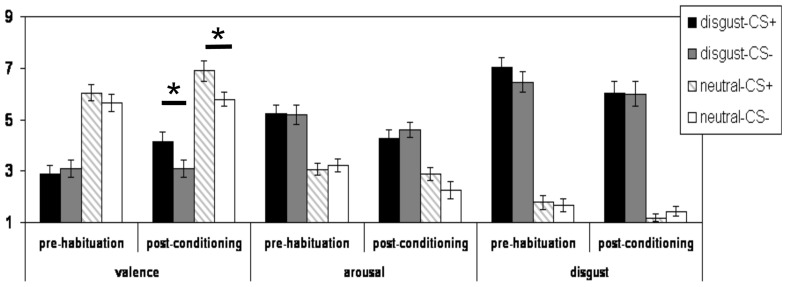
**Mean subjective valence, arousal, and disgust ratings (and standard errors of the mean) of for the CS+_disg_, the CS-_disg_, the CS+_con_, and the CS-_con_ before the habituation phase and after the conditioning phase.** ^*^indicates *p* < 0.05.

Regarding the analyses of the valence ratings, the three-way interaction did not reach the significance level (*p* > 0.15). A significant two-way interaction effect of the factors “phase” × “reward learning” [*F*_(1, 27)_ = 6.44; *p* = 0.017] was observed. Both CS+ (i.e., CS+_disg_ and CS+_con_) were rated more positively after conditioning than before the habituation phase as compared to their corresponding CS− (see Figure 1). Moreover, a highly significant main effect of “CS-emotion” was found [*F*_(1, 27)_ = 65.56; *p* < 0.001]: the disgust pictures were rated as significantly more unpleasant as compared to the control pictures (see Figure 1). In addition, a significant main effect of the factor “phase” was observed [*F*_(1, 27)_ = 7.49; *p* < 0.011], with overall more positive valence ratings after conditioning compared to before the habituation phase.

*Post hoc* analyses (with Bonferroni correction) of the valence ratings confirmed that before the habituation phase, there was no significant difference in valence ratings between CS+_disg_ and CS−_disg_ (*p* > 0.2). Likewise, CS+_con_ and CS−_con_ were not rated differently (*p* > 0.2) before the habituation phase. After conditioning, significant differences emerged for the comparison CS+_disg_ vs. CS−_disg_ [*t*_(27)_ = 3.17; *p* = 0.004] and the comparison CS+_con_ vs. CS−_con_ [*t*_(27)_ = 3.35; *p* = 0.002]. Both CS+ were rated more positively as compared to the corresponding CS−.

Regarding the analyses of the arousal ratings, no significant interaction effects were observed (all *p* > 0.05). Yet, a significant main effect of the factor “CS-emotion” was detected [*F*_(1, 27)_ = 37.72; *p* < 0.001]: the disgust pictures were rated as significantly higher arousing as compared to the control pictures. Moreover, a significant main effect of the factor “phase” was observed [*F*_(1, 27)_ = 7.93; *p* = 0.009]: overall, arousal ratings were lower after the conditioning as compared to before the habituation phase.

Regarding the analyses of the disgust ratings, no significant interaction effects were observed (all *p* > 0.10). We again observed a significant main effect of the factor “CS-emotion” [*F*_(1, 27)_ = 161.91; *p* < 0.001]: as expected, the disgust CS received higher disgust ratings. Moreover, we observed a significant main effect of the factor “phase” [*F*_(1, 27)_ = 7.12; *p* < 0.012].

Next, we correlated the differential rating scores of the comparison CS+_disg_ to CS−_disg_ [i.e., (CS+_disg_ to CS−_disg_)_pre-habituation_—(CS+_disg_ to CS−_disg_)_post-conditioning_] with the disgust sensitivity scores. We found a significant positive correlation of disgust sensitivity with the differential arousal ratings (*r* = 0.44; *p* = 0.021). The other ratings scales were not significantly correlated with the disgust sensitivity scores (*p* > 0.40).

### fMRI

#### Manipulation check

As a manipulation check, we first analyzed the contrast UCS > non-UCS in the conditioning phase. As expected, the contrast UCS > non-UCS revealed strong whole-brain as well as ROI-activation (e.g., bilaterally in the NAcc). Statistical parameters and coordinates of the significant results of the exploratory whole-brain as well as the ROI analyses for this contrast can be found in Table 1.

**Table 1:** **Correction volume, structures, cluster sizes (*k*), coordinates of peak voxels, *t*-values, and *p*_corr_–values (FWE correction) for the contrast UCS > non-UCS (from the conditioning phase)**.

**Contrast: UCS > non-UCS**								
**Correction volume**	**Structure**	**Side**	***k***	***x***	***y***	***z***	***t*_max_**	***p*_corr_**
Whole-brain	Occipital fusiform gyrus	R	5745	30	−76	−8	20.05	<0.001
	Precentral gyrus	R	406	42	8	28	11.13	<0.001
	Frontal orbital cortex	R	300	33	26	−5	10.68	<0.001
	Thalamus	R	242	21	−31	1	10.45	<0.001
	Paracingulate gyrus	R	307	3	23	43	10.18	<0.001
	Precentral gyrus	L	242	−42	2	34	9.67	<0.001
	Frontal orbital cortex	L	196	−30	23	−8	8.71	<0.001
	Frontal pole	R	72	42	59	1	7.98	<0.001
	Posterior cingulate gyrus	L	53	−3	−25	28	7.89	0.001
	Superior frontal gyrus	L	13	−18	17	67	7.50	0.002
	Frontal pole	R	75	51	47	19	7.03	0.005
	Thalamus	L	11	−9	−1	13	6.83	0.009
	Frontal pole	R	21	27	38	−20	6.61	0.015
	Superior frontal gyrus	L	5	−9	5	76	6.29	0.032
ROI	OFC	R	315	33	26	−5	10.68	<0.001
	Insula	R	147	33	26	−2	10.65	<0.001
	OFC	L	293	−30	23	−8	8.71	<0.001
	Insula	L	156	−30	23	−2	8.70	<0.001
	ACC	R	293	3	23	34	7.74	<0.001
	NAcc	L	22	−12	11	−11	5.13	<0.001
	Amygdala	L	16	−21	−4	−11	4.72	0.002
	Amygdala	R	5	30	2	−17	3.90	0.014
	NAcc	R	3	12	14	−5	3.64	0.009

*Labeling of the results from the exploratory whole-brain analyses was performed using the probabilistic Harvard-Oxford Cortical and Subcortical Atlases. The significance threshold was p_corr_ < 0.05. The cluster forming threshold was p_corr_ < 0.05 and k ≥ 5 voxel for the whole-brain analyses and p_uncorr_ < 0.001 and k ≥ 5 for the ROI analyses. All coordinates are given in MNI space*.

Next, as a second manipulation check, we analyzed the contrast of the two disgust vs. the two control pictures in the habituation phase. The exploratory whole-brain analyses revealed stronger activity to the disgust pictures. Two bilateral clusters of activation, both ranging from primary visual cortex areas into the posterior fusiform gyrus peaking in the left occipital pole (*x* = −24, *y* = −94, *z* = −2; *t* = 13.47; *k* = 893; *p*_FWE_ < 0.001) and the right lingual gyrus (*x* = 27, *y* = −49, *z* = −8; *t* = 12.53; *k* = 834; *p*_FWE_ < 0.001), respectively, were observed. Further, ROI analyses revealed significant bilateral OFC (right: *x* = 27, *y* = 32, *z* = −14; *t* = 5.29; *p*_FWE_ = 0.003; left: *x* = −33, *y* = 23, *z* = −8; *t* = 4.88; *p*_FWE_ = 0.007), right amygdala (*x* = 24, *y* = −7, *z* = −14; *t* = 4.93; *p*_FWE_ = 0.002), as well as left insula activation (*x* = −33, *y* = −20, *z* = −5; *t* = 5.08; *p*_FWE_ = 0.004).

#### Analyses of conditioned responses

**Figure 2:**
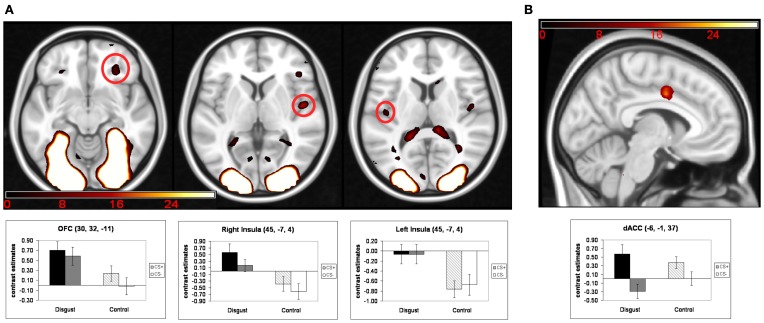
**Results of the ANOVA of conditioned responses: (A) main effects of the factor “CS-emotion”; (B) main effects of the factor “reward learning”.** Mean contrast estimates (and standard errors of the mean) of the CS in the respective peak voxels are illustrated in the bar graphs. The threshold for displaying the images is set at *p*_uncorr_ < 0.005 and *k* > 5 voxels.

Turning to the main analysis of hemodynamic activity during the conditioning phase, conditioned responses (CR) were analyzed using a 2 (“CS-emotion”: disgust vs. control) × 2 (“reward learning”: CS+ vs. CS−) ANOVA. The exploratory whole-brain analyses did not yield a significant interaction effect or a significant main effect of reward learning. We found significant whole-brain main effects for the factor “CS-emotion” (i.e., disgust vs. control), which were similar to the results of the contrast disgust > control in the habituation phase. Again, strong whole-brain effects were found in visual areas, with peaks in the occipital pole and in the thalamus (see Figure 2A and Table 2).

**Table 2:** **Correction volume, structures, side, cluster sizes (*k*), coordinates of peak voxels, peak *F*-values, and p_corr_-values (FWE correction) for the results of the ANOVA of conditioned responses**.

**Contrast**	**Correction method**	**Structure**	**Side**	***k***	***x***	***y***	***z***	***F*_max_**	***p*_corr_**
Main effect of CS-emotion	ROI	OFC	R	3	30	32	−11	16.92	0.014
	ROI	Insula	L	2	−42	−13	10	15.17	0.027
	ROI	Insula	R	2	45	−7	4	19.50	0.005
	whole-brain	Occipital pole	L	1296	−15	−97	−5	271.66	<0.001
	whole-brain	Occipital pole	R	1291	18	−94	1	149.59	<0.001
	whole-brain	Lingual gyrus	L	12	−6	−88	−19	29.99	0.012
	whole-brain	Thalamus	R	3	12	−31	16	26.84	0.031
Main effect of reward learning	ROI	dACC	L	23	−6	−1	37	20.94	0.005
Interaction CS-emotion × reward learning	No significant effects								

*Labeling of the results from the exploratory whole-brain analyses were performed using the probabilistic Harvard-Oxford Cortical and Subcortical Atlases. The significance threshold was p_corr_ < 0.05 (FWE-corrected). The cluster forming threshold was p_corr_ < 0.05 and k ≥ 5 voxel for the whole-brain analyses and p_uncorr_ < 0.001 and k ≥ 5 for the ROI analyses. All coordinates are given in MNI space*.

Next, we analyzed our a priori ROI. Again, no significant interaction effects were detected. Moreover, ROI analyses revealed a main effect of “reward learning” in the dACC (see Figure 2B and Table 2). Inspection of beta-estimates revealed that this was due to larger dACC activity in response to the two CS+ as compared to the two CS−. ROI analyses of the main effect “CS-emotion” showed significant effects bilaterally in the insula and in the right OFC (see Figure 2A). Assessment of beta-estimates confirmed that these effects were due to larger responses to the disgust than to the control pictures. All statistical parameters and MNI-coordinates regarding the results of the ANOVA can be found in Table 2.

**Figure 3:**
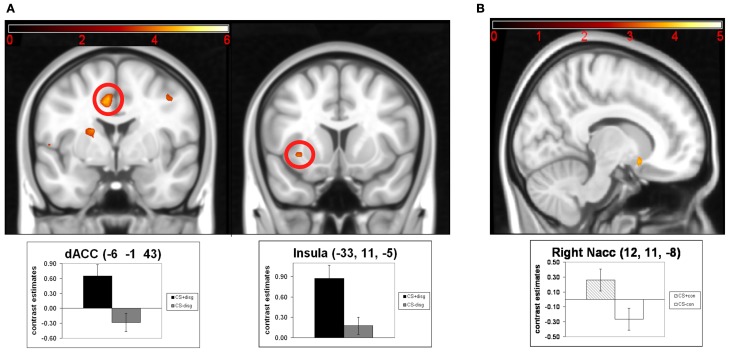
**Results of the paired *t*-tests of the separate analysis of conditioned responses in the counterconditioning and the control conditions: (A) neuronal activations for the contrast CS+_disg_ minus CS-_disg_; (B) neuronal activations for the contrast CS+_con_ minus CS-_con_.** Mean contrast estimates (and standard errors of the mean) of the CS in the respective peak voxels are illustrated in the bar graphs. The threshold for displaying the images is set at *p*_uncorr_ < 0.005 and *k* > 5 voxels.

Next, we analyzed following contrasts using paired *t*-tests: CS+_disg_ > CS−_disg_ and CS+_con_ > CS−_con_. The contrast CS+_disg_ > CS−_disg_ revealed no significant effects in the exploratory whole-brain analyses. The ROI analyses (see Figure 3A) in the same contrast showed significant activation in the dACC (*x* = −6, *y* = −1, *z* = 43; *t* = 4.67; *p*_FWE_ = 0.014) and the left insula (*x* = −33, *y* = 11, *z* = −5; *t* = 4.11; *p*_FWE_ = 0.031). Finally, in the contrast CS+_con_> CS−_con_ exploratory whole-brain analyses again did not show significant effects. Subsequent ROI analyses (see Figure 3B) revealed significant differentiation in the right NAcc (*x* = 12, *y* = 11, *z* = −8; *t* = 3.72; *p*_FWE_ = 0.007).

### Correlational analyses

Regarding the correlational analyses, we found no significant results for the correlation of the hemodynamic activity in the contrast CS+_disg_> CS−_disg_ with the subjective valence ratings. Activity in the dACC exceeded the significance threshold only marginally (*x* = −6, *y* = −1, *z* = 37; *t* = 4.03; *p*_FWE_ = 0.064; *k* = 8). Interestingly, this was the same voxel which showed the main effect of conditioning in the ANOVA of conditioned responses.

Regarding the correlational analyses of the contrast CS+_con_ > CS−_con_ with the subjective valence ratings, we did not find any significant results.

Regarding the correlational analyses of disgust sensitivity with the contrast CS+_disg_ > CS−_disg_, we again did not observe significant findings.

## Discussion

In the present study, we investigated whether activity in brain areas that have been previously associated with disgust responses and/or to reward learning and anticipation are altered by a counterconditioning procedure. Clear evaluative conditioning effects were found with regard to the subjective valence ratings, however, no interaction effect was observed, i.e., the increase in valence was the same for the CS+_disg_ and the CS+_con_. Regarding the hemodynamic responses, analyses of variance revealed significant main effects of reward learning and anticipation as well as a main effect of the emotional content of the CS pictures. In detail, higher responses to the two CS+ were found in the dACC and stronger insula and OFC activity was observed in response to the disgust as compared to the control pictures. Again, no interaction effect (i.e., reward learning x CS-emotion) was observed. Taken together, the results suggest that affective processing of disgust stimuli and reward learning and anticipation may not influence each other. However, subsequent analyses revealed higher insula and dACC activity in the contrast CS+_disg_ > CS−_disg_ implicating a potential role of these structures in the counterconditioning of disgust responses.

In line with previous findings (Eifert et al., 1988; Kerkhof et al., 2011), analyses of subjective ratings revealed conditioning effects for the valence ratings as indicated by the significant phase × CS-type interaction. Although the emotional content of the CS clearly led to highly significant overall differences between the neutral and the disgust pictures on each of the three rating dimensions, it did not differentially influence conditioning of the disgust and the control condition. Interestingly, in contrast to the valence ratings, no differential changes in subjective disgust ratings were observed between CS+_disg_ and disgust CS−_disg_. This result implies that although the CS+_disg_ was evaluated more pleasantly than the disgust CS−, its disgust-inducing properties were not subject to any changes. This dissociation of the two rating dimensions raises some interesting implications. First, it corresponds to the finding that on the subjective level disgust responses are very resistant to extinction (Rozin and Fallon, 1987; Smits et al., 2002), even when induced through second-order conditioning (Olatunji, 2006; Klucken et al., 2012a). On the other hand, the dissociation of the two rating dimensions disgust and valence may point to an affective conflict created through the negative affect of the disgust pictures and the positive anticipatory affect of the appetitive UCS. Nevertheless, it has to be noted that an overall decline in subjective ratings was observed, which was most likely due to habituation.

Concerning hemodynamic activity, the comparison of the disgust with the control pictures during the habituation phase as well as the main effect of CS-emotion in the analysis of conditioned responses showed stronger activity to the disgust pictures in the insula, the OFC, the amygdala, the thalamus, and the extended occipital cortex. These findings are well in line with previous studies on disgust perception and processing (for review see Cisler et al., 2009; Olatunji et al., 2010), in which altered hemodynamic activity of the insula, the OFC, the amygdala, and the occipital cortex in response to disgust-relevant stimuli have been repeatedly reported (e.g., Schienle et al., 2002b; Wright et al., 2004; Caseras et al., 2007; Jabbi et al., 2008; Schäfer et al., 2009). Underlining the importance of the insula for disgust processing, a recent meta-analysis found that the insula reliably differentiated disgust from all other emotional states (Vytal and Hamann, 2010). Activity in the OFC in response to disgust stimuli has been reported to correlate with the trait disgust sensitivity (Schienle et al., 2006; Schäfer et al., 2009), although this finding could not be replicated in the present study. Moreover, OFC activity has been found to be higher in response to contamination-related as compared to mutilation-related disgust stimuli, which is in line with our findings (Schienle et al., 2006).

In addition to the main effect of CS-emotion, we observed a main effect of reward learning and anticipation in the dACC. Stronger responses to the CS+ as compared to the corresponding CS- were observed in both conditions. Activity in the dACC is a commonly observed result in tasks that involve cues that signal an affective outcome (Martin-Soelch et al., 2007; Sehlmeyer et al., 2009). Our finding suggests that dACC activity was not affected by the emotional properties of the disgust CS. At first glance, this result is in contrast to the consistent finding of dACC activity in tasks that involve conflict or other kinds of error processing, including emotional conflict (Botvinick, 2007; Taylor et al., 2007). However, alternative views on dACC function propose that the dACC activity triggered by the enhanced cognitive load during conflict processing may act as a learning signal conveying adaptive control (Botvinick, 2007; Shackman et al., 2011). Moreover, the result is in line with the wealth of evidence that links the dACC to reward learning and anticipation (Martin-Soelch et al., 2007; Haber and Knutson, 2010). It also fits to the view that the dACC codes for the anticipated reward value considering that CS+_disg_ as well as control CS+_con_ predicted the same UCS (O'Doherty, 2004).

Although we observed effects of reward learning and anticipation in both conditions, the employed counterconditioning procedure did not lead to significant differences between the disgust and the control CS in areas associated with the processing of reward learning and anticipation and/or with disgust processing. This pattern of results was observed in the hemodynamic as well as the subjective responses. This result may imply that the negative affect generated by the disgust pictures and the positive affect generated by the anticipation of the monetary UCS are processed separately in the brain and the conditioned responses were based on specific visual features of the stimuli. In the subsequent analyses, enhanced dACC and insula activity to the CS+_disg_ as compared to the CS−_disg_ was observed. This points to potential roles for these structures in the alteration of disgust responses through counterconditioning. At least, the findings reflect the increased salience that the CS+_disg_ obtained through the counterconditioning procedure (cf. Menon and Uddin, 2010).

Contrary to our expectations, we did not find effects of the counterconditioning procedure in NAcc and amygdala (i.e., main or interaction effects). Regarding the NAcc, we observed a differentiation in the analysis of the control condition only (i.e., in the contrast CS+_con_ > CS−_con_). This result is in accordance with the well-documented role of the NAcc in reward learning and anticipation (e.g., Kirsch et al., 2003 see Martin-Soelch et al., 2007; Haber and Knutson, 2010). The absence of differential NAcc activity in the contrast CS+_disg_ > CS−_disg_ may indicate an influence of CS valence on hemodynamic activity in this brain area, which may have slowed down the learning rate of the prediction error signal that has been associated with NAcc activity in the past (Schultz, 1997, 2002; McClure et al., 2003; O'Doherty et al., 2003). However, this assumption remains speculative and must be treated with caution. In addition, other than expected, we also did not find amygdala effects. This could be due to habituation (cf. LaBar et al., 1998) or ceiling effects of the negative pictures. Still, studies investigating reward learning and anticipation have only rarely reported effects of the amygdala (Martin-Soelch et al., 2007).

Taken together, the observed results allow the interpretation that the employed counterconditioning procedure did not directly affect disgust responding, as indexed by the lack of change in disgust ratings and the absence of interaction effects in brain regions associated to disgust responding, such as the OFC and the insula. Previous studies have demonstrated that disgust responses can be reduced through exposure (e.g., Smits et al., 2002; Olatunji et al., 2012; Viar-Paxton and Olatunji, 2012), which was also observed in this study. However, this (within-session) reduction is context dependent (Viar-Paxton and Olatunji, 2012) and remarkably smaller compared to reductions in fear responses through exposure (Smits et al., 2002; Olatunji et al., 2012). Moreover, subjective and neuronal disgust responses remain relatively stable between sessions, indicating little between-session reduction of disgust responding (Stark et al., 2004). These findings are paralleled by reports on prolonged extinction of disgust-relevant conditioned responses (Olatunji et al., 2007; Klucken et al., 2013). Thus, the lack of change in subjective and hemodynamic disgust responding in this study is in line with the view that disgust responses are particularly difficult to modify (Rozin and Fallon, 1987; Olatunji et al., 2010; Mason and Richardson, 2012). Furthermore, specific stimulus attributes, such as the nature and the similarity of the disgust stimuli (i.e., faeces vs. other kinds of disgust stimuli, e.g., rotten bodies, cockroaches, cf. Rozin and Fallon, 1987) may have also influenced the observed results in the current study. Nevertheless, the lack of effects in disgust responding does not necessarily imply that the disgust responses were totally unaffected by the counterconditioning procedure. In the case of fear, it has been demonstrated that the level of fear during exposure is not predictive of the level of fear at re-exposure (see review by Craske et al., 2008). Moreover, it has been shown that the valence difference between CS+ and CS− is predictive for the amount of behavioral reinstatement in a later test phase in a fear conditioning experiment (Dirikx et al., 2004, 2007). However, it is an open question whether these findings hold for the counterconditioning of disgust responses.

We would finally like to address some potential shortcomings of the present study. First, the observed effects may only hold for disgust stimuli from the category contamination / body secretion (cf. Rozin and Fallon, 1987). It is unclear whether other types of disgust stimuli could have led to different results. Second, it is possible that a closer fit of CS and UCS could lead to stronger effects of the counterconditioning procedure, for example the usage of pleasant odor as UCS. In addition, we cannot exclude that conditioning was influenced by the repeated presentation and the relatively long presentation time of the stimuli, which clearly created habituation effects. Finally, since our study investigated healthy subjects, it is unclear how exaggerated disgust responses in subjects suffering from psychiatric disorders are affected by counterconditioning.

In conclusion, the observed effects in the behavioral and the fMRI data suggest that the emotional content of the disgust pictures did not differentially alter the magnitude of the conditioned responses. The results imply that disgust responses and reward learning and anticipation may not influence each other. Nevertheless, the separate analysis of the counterconditioning condition indicates that the dACC and the insula may play a role in the alteration of disgust responses through counterconditioning. In sum, the results of this first study on the neuronal correlates of counterconditioning in humans add to the ongoing debate on the transfer of neuronal foundations of emotional learning processes to behavioral treatment strategies and add to a more sophisticated understanding of human emotions.

### Conflict of interest statement

The authors declare that the research was conducted in the absence of any commercial or financial relationships that could be construed as a potential conflict of interest.
